# Electrically conductive scaffolds mimicking the hierarchical structure of cardiac myofibers

**DOI:** 10.1038/s41598-023-29780-w

**Published:** 2023-02-17

**Authors:** Arsalan Ul Haq, Luca Montaina, Francesca Pescosolido, Felicia Carotenuto, Federica Trovalusci, Fabio De Matteis, Emanuela Tamburri, Paolo Di Nardo

**Affiliations:** 1 Department of Clinical Sciences and Translational Medicine, University of Rome “Tor Vergata”, Via Montpellier 1, 00133 Rome, Italy; 2 Interdepartmental Research Centre for Regenerative Medicine (CIMER), University of Rome “Tor Vergata”, Via Montpellier 1, 00133 Rome, Italy; 3 Department of Chemical Science and Technologies, University of Rome “Tor Vergata”, Via Della Ricerca Scientifica, 00133 Rome, Italy; 4 Department of Enterprise Engineering “Mario Lucertini”, University of Rome “Tor Vergata”, Via del Politecnico 1, 00133 Rome, Italy; 5 Department of Industrial Engineering, University of Rome “Tor Vergata”, Via del Politecnico 1, 00133 Rome, Italy

**Keywords:** Materials science, Biomaterials, Materials chemistry, Translational research

## Abstract

Electrically conductive scaffolds, mimicking the unique directional alignment of muscle fibers in the myocardium, are fabricated using the 3D printing micro-stereolithography technique. Polyethylene glycol diacrylate (photo-sensitive polymer), Irgacure 819 (photo-initiator), curcumin (dye) and polyaniline (conductive polymer) are blended to make the conductive ink that is crosslinked using free radical photo-polymerization reaction. Curcumin acts as a liquid filter and prevents light from penetrating deep into the photo-sensitive solution and plays a central role in the 3D printing process. The obtained scaffolds demonstrate well defined morphology with an average pore size of 300 ± 15 μm and semi-conducting properties with a conductivity of ~ 10^–6 ^S/m. Cyclic voltammetry analyses detect the electroactivity and highlight how the electron transfer also involve an ionic diffusion between the polymer and the electrolyte solution. Scaffolds reach their maximum swelling extent 30 min after immersing in the PBS at 37 °C and after 4 weeks they demonstrate a slow hydrolytic degradation rate typical of polyethylene glycol network. Conductive scaffolds display tunable conductivity and provide an optimal environment to the cultured mouse cardiac progenitor cells.

## Introduction

Biological tissues are usually categorized by the cell types embedded in their texture, the expression of different molecules contributing to their machinery and the families of factors secreted out in various steps of their differentiation. However, they are also traversed by feeble electric currents crucial for their intercellular communication and functioning^1^. In the myocardium and nerves, these basal currents are superimposed by self-generated cyclic electric waves able to spawn signals and mechanical forces that, through adjacent cells, travel to the most peripheral regions of the body. In this context, the function of the myocardial tissue is modulated by the unique mechanical properties and anisotropic structure of the cardiac tissue in which the wide three-dimensional extracellular matrix (ECM) network orients the cardiomyocytes, mechanically couples them ensuring their electrical connectivity, and provides elastic support during ventricular contraction. The myocardial fiber orientation varies transmurally throughout the ventricular wall. These fibers run in the right-handed helix direction at the sub-endocardial region, pass circumferentially through the mid-wall and turn to the left-handed helix direction at the sub-epicardial region contributing significantly to the cardiac pumping^2,3^. Traumatic events and degenerative diseases, among others, often lead to unrepairable damages to this admirable bioarchitecture owing to the poor cardiac innate regeneration capability^4^. Injured areas are replaced with collagen-rich scar tissue that warps the ventricular geometry and obstructs the regular flow of electrical signals leading to arrhythmias and heart failure in the long run^5^.

In the past decades, the progress in biological sciences, engineering, materials science, and advanced micro/nano manufacturing techniques have suggested the possibility to repair injured ventricular regions by fabricating and implanting stripes of healthy myocardium. To this end, a multidisciplinary approach (tissue engineering) has been employed to match the complexity of the myocardial tissue bioarchitecture and function. In a typical tissue engineering experiment, stem cells are seeded in a biocompatible polymeric scaffold vaguely emulating the ECM of the tissue. The scaffold is usually composed of natural or synthetic biomaterial or a combination of both (scaffold) and using this approach several artificial heart-like tissues have been engineered and implanted in vivo^6–11^. However, despite extraordinary efforts worldwide, the results have not yet been adequate to envision clinical use^12,13^. The cause of this failure, among others, can mostly be found in scaffolds inadequately mimicking the tissue bioarchitecture^14^. Initially, the scaffolds were merely intended as a mechanical support for the cultured cells to grow and proliferate. Later, it was revealed that cellular differentiation can be enhanced via specific physical, chemical, mechanical and biological properties of the scaffold while waiting for the cells to secrete their own ECM.

Extensive literature could be found on scaffolds demonstrating a few or all these properties^15–18^. However, two major issues have not been adequately addressed properly in otherwise non-electrically conductive scaffolds currently in use. The first is how conferring upon the scaffold the ability to induce the proper cardiomyocytes orientation. The second is how creating an electroconductive environment may improve intercellular communication and synchronization among immature cardiac stem cells suffering from reduced conductivity due to still poor organization of the gap junctions.

The gap junctions are cell membrane structures that allow the rapid exchange of ions, which is necessary for the efficient propagation of action potentials through the heart wall and the spreading of the signaling molecules. However, the gap junctions in human cardiomyocytes are distributed throughout their entire cell surface during the neonatal stages and reach their final maturation and polarization into the intercalated discs only when human beings are 6 years old^19^. A similar distribution was observed in murine and canines where the gap junctions reached their maturity and polarization only when the animals were 90 days old^20^. These data suggest that myocardial electroconductivity is associated with two different electrical grids: the first is active during early life, is based on the ECM and greatly contributes to signaling the developing immature cells. While the second is typically based on the mature cardiomyocytes, conveys the stimulus for contraction and has a poor effect on the cell phenotype when established.

Starting from previous studies on innovative methods to prepare biomaterials and biosystems^21–23^, the main goal of the present study was to develop electrically conductive scaffolds mimicking the hierarchical orientation of cardiac myofibers using 3D printing projection micro-stereolithography approach from a technical perspective.

The conductivity was achieved by blending the conductive polymer polyaniline (PANI) with polyethylene glycol diacrylate (PEGDA) hydrogels to make a conductive ink for 3D printing. The obtained scaffolds demonstrated semi-conducting properties (~ 10^–6^ S/m) with an average pore size of 300 ± 15 μm. The conductivity can be tailored easily by controlling the concentration of PANI in the precursor solution. The viability of the mouse cardiac progenitor cells cultured on conductive scaffolds was comparable to cells cultured on non-conductive scaffolds.

## Materials and methods

All reagents were purchased from Merck KGaA except when mentioned otherwise and were used as received.

### Synthesis of PANI

PANI was synthesized following a previously optimized protocol^24^. In brief, after distillation under reduced pressure, 5 mmol of aniline (Fluka) was added to a 1 M HCl solution containing an appropriate amount of sodium dodecyl sulphate (SDS) and sonicated for 30 min. In parallel, 5 mmol of ammonium persulphate was added to another 1 M HCl solution. This mixture was slowly added to the above aniline mixture, cooled down to 0–5 °C and left on stirring for 6 h. During this time, a dark green PANI precipitate started to form indicating the polymerization of aniline into PANI. After complete precipitation, PANI was filtered out and washed thoroughly with 1 M HCl solution then with water, and ethanol. After thorough washing, the slurry was dried overnight at 50 °C under the vacuum. The obtained PANI powder was then grounded in a mortar and pestle to obtain a fine particle size.

### Preparation of the conductive ink for 3D printing

The precursor solution for photo-polymerization was a blend of PEGDA (MW 575 Da) as the photo-sensitive polymer, curcumin extracted from *Curcuma longa* as the light absorbing dye, and bis(2,4,6-trimethylbenzoyl)-phenylphosphineoxide (also known as Irgacure 819) as the photo-initiator. Curcumin and Irgacure were dissolved in the PEGDA:Ethanol 3:1 volume ratio solution to obtain the precursor system (Table 1). The flask covered with aluminum foil was left overnight for stirring in a dark environment. Later, a proper amount of the synthetized PANI powder was then dispersed in the precursor solution by sonication and magnetic stirring. This process was repeated each time to obtain photocurable inks with different amounts of dispersed PANI.

**Table 1:** Experimental parameters for preparing and printing conductive inks.

Sample name	Irgacure 819 (% w/v)	Curcumin (% w/v)	PANI (% w/v)	Exposure time per impression (seconds)
PEGDA	1.83	0.016	0.0	6
0.3% PANI	1.83	0.016	0.3	6
0.6% PANI	1.83	0.016	0.6	8
0.9% PANI	1.83	0.016	0.9	10
1.5% PANI	1.83	0.016	1.5	15
2.0% PANI	1.83	0.016	2.0	20
2.5% PANI	1.83	0.016	2.5	> 20
3.0% PANI	1.83	0.016	3.0	> 20

### Electroconductive scaffold fabrication

**Figure 1:**
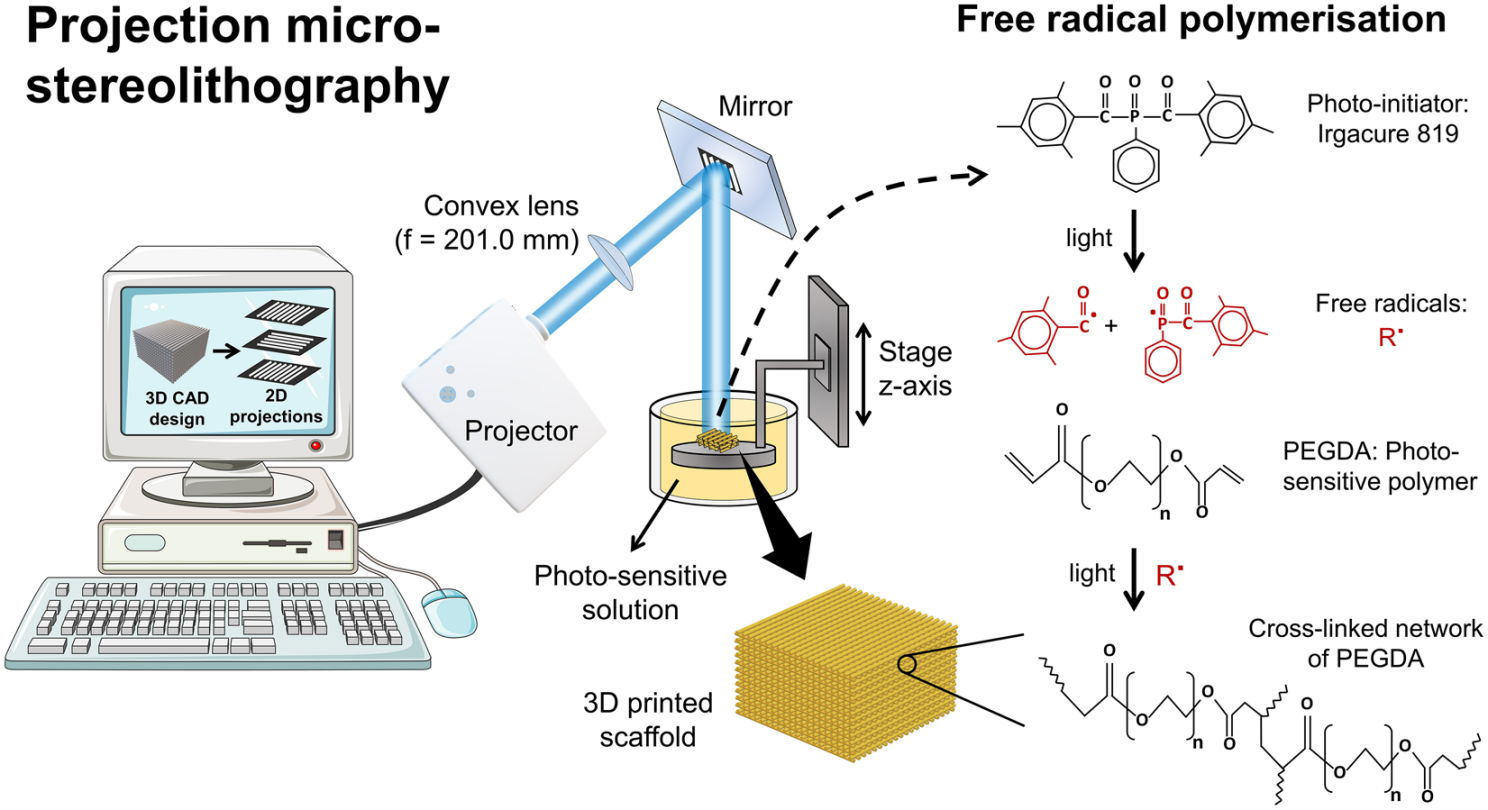
Scheme of the custom-built 3D printing micro-stereolithography setup. The scaffold is designed using AutoCAD and the 3D geometry is virtually sliced into 2D projections/impressions that act as digital photomask. The projector shines these impressions on a mirror which are then reflected onto the surface of photo-sensitive solution. Only the lit area of the solution is crosslinked via the photo-polymerisation reaction printing a solid layer. The stage is moved down by a few microns and the fresh solution covers this printed layer. Again, the solution is lit with another impression to deposit a second layer on top of the first layer. In this way, a 3D scaffold is fabricated by depositing 2D layers on top of each other. Some images were obtained from the Servier Medical Art under the CC-BY 3.0 license.

Projection micro-stereolithography (PµSL) was used to fabricate the scaffolds as shown in Fig. 1. The whole PμSL setup was mounted on an optical breadboard. To fabricate a scaffold, first, it was designed using AutoCAD software version 2016 for Windows (https://www.autodesk.com/products/autocad) after considering optimal design parameters such as the shape, pore size, geometry, dimensions etc. A stereolithography file (.stl) was exported and was used to virtually slice the 3D geometry into 2D projections using the Slic3r software version 1.3.0 for Windows (https://slic3r.org/). These sliced projections were used as digital impressions and they can also be created in PowerPoint software. An overhead projector (Acer X1385WH) containing a high-pressure mercury arc lamp with a luminous flux of 3400 lumens was used as the source of visible light.

The 3-axis movement of the stage was controlled manually via the micrometers mounted on it and they could move the stage by a minimum distance of 10 μm along each axis. Before starting the printing process, the stage was submerged by 100 μm in the solution corresponding to the thickness of each printed layer. Then from the projector, each impression was focused on a mirror via a convex lens (focal length = 201.0 mm). It was then reflected on the surface of the photosensitive solution in which the stage was already dipped. The area of the photosensitive solution that was lit with an impression crosslinked the solution via the free radical polymerization reaction, thus producing a 2D layer. The thickness of this printed layer was 100 μm corresponding to how deep the stage was initially submerged in the solution. After the deposition of first layer, the stage was submerged again in the solution by 100 µm and the fresh solution covered this printed layer. It was then lit with the second impression thus depositing the second layer with 100 μm thickness on top of the first layer. The continuous deposition of layers on top of each other produced a 3D scaffold with controlled thickness of each layer. The PEGDA matrix was produced when the solution was lit with a solid impression (for example a full circle) contrary to the fibrous impressions. This in turn produced a solid layer without pores, via the same free radical polymerization reaction described in Fig. 1.

## Characterizations techniques

### Morphological and structural analyses

Light absorbance spectra were obtained in the range of 300–800 nm using spectrophotometer (Perkin Elmer Lambda 750). Scanning electron microscopy (SEM) was performed to analyse the morphology of the scaffolds. Scaffolds’ surface was scanned at different voltages (5, 10, 15 kV) and magnifications using HITACHI TM 4000 scanning electron microscope. Infrared spectroscopy was performed using ThermoFisher Scientific™ Nicolet™ iS50 FTIR spectrometer with diamond crystal ATR integrated module. The scan was carried out between 4000 and 500 cm^−1^ in the absorbance mode.

### Electrical conductivity measurements

The bulk conductivity of the scaffolds was evaluated via two probe conductivity measurements at room temperature using Keithley 2700 Multimeter. Scaffolds (a few microns thin) were placed between two steel probes (1.5 mm radius) to measure their electrical resistance in a time sweep mode. The resistance scanned at various time points was recorded using the LabVIEW software. The electrical conductivity was calculated using Eq. (1):

1 $$\sigma = \frac{l}{{\pi r}^{2}.R}$$

where σ = Electrical conductivity (S/m), l = Thickness of the scaffold (m), r = Radius of the steel probe (m), R = Average electrical resistance (Ω).

### Cyclic voltammetry tests

The electrochemical response was evaluated using cyclic voltammetry (CV) in a standard one-compartment three-electrode cell using a Palm Sense compact electrochemical workstation. The CV curves were obtained in the + 0.4 V to + 0.9 V range at different scan rates (10–18 mV/s). A saturated calomel electrode (SCE) and a platinum foil were used as the reference and counter electrode, respectively. The working electrode was realized by providing a platinum contact to the back side of the investigating scaffolds.

### Swelling degree analysis

The swelling degree was measured to evaluate the swelling limit of the scaffolds under physiological conditions (pH = 7.4, 37 °C). Scaffolds were immersed in 1X PBS and were kept at 37 °C. The swelling degree was determined by measuring the mass difference between the dry scaffold and the wet scaffold at different time intervals using the following Eq. (2):

2 $$Sweeling \; degree (\%)= \frac{({M}_{w}-{M}_{d})}{{M}_{d}}\times 100$$

where M_w_ = Mass of the wet scaffold at time t, M_d_ = Mass of the dry scaffold.

### Hydrolytic degradation

Degradation kinetics were monitored to find out the degradation rate of different scaffolds. For this purpose, scaffolds were immersed in the 1X PBS at 37 °C for 4 weeks. After each week, how much mass each scaffold lost was calculated by measuring the difference between the mass of the fully swollen scaffold and the mass of the scaffold after each week using the following Eq. (3):

3 $$Mass \; loss (\%)= \frac{({M}_{s}-{M}_{t})}{{M}_{s}}\times 100$$

where M_s_ = Mass of the fully swollen scaffold, M_t_ = Mass of the scaffold at time t.

### Cell culture and viability

Mouse cardiac progenitor cells (mCPCs) were used to test the scaffold biocompatibility. The cells were isolated from the hearts of 6 weeks old C57/BI mice as previously described^25^. Cells were cultured in DMEM (Gibco) containing 10% FBS (Gibco), 1% penicillin–streptomycin, and 1% L-Glutamine (Sigma Aldrich). Before cell seeding, the scaffolds were soaked in 70% ethanol solution for 1 h for sterilization. After this, they were dried in a sterile ventilated biological hood and placed in a 24-well plate. The scaffolds were then washed with sterile PBS and equilibrated with DMEM containing 20% FBS for 24 h at 37 °C to improve the cell adhesion.

Cells were seeded at the density of 2 × 10^4^ cells/cm^2^ and incubated in DMEM containing 10% FBS at 37 °C. The cells placed in wells without scaffolds were considered as the control group. After 120 h, both adherent and suspended cells of each well were harvested and stained with 0.4% trypan blue assay to assess the viability (Sigma-Aldrich, Milan, Italy). The live and dead cells were counted under an inverted optical microscope carried out in triplicate and repeated three times.

### Statistical analysis

The statistical analysis was performed using GraphPad Prism (GraphPad Software, San Diego, CA, USA, https://www.graphpad.com). Data from six independent experiments were quantified and analyzed for each variable. The two-tailed t-test with equal variance was used to compare the means as well as one way ANOVA test. The threshold for statistical significance was set at *P* < 0.05 and the standard deviations (S.D) of the mean values were calculated for each type of sample. The data are presented as mean ± S.D.

## Results

### UV–Visible spectroscopy

**Figure 2:**
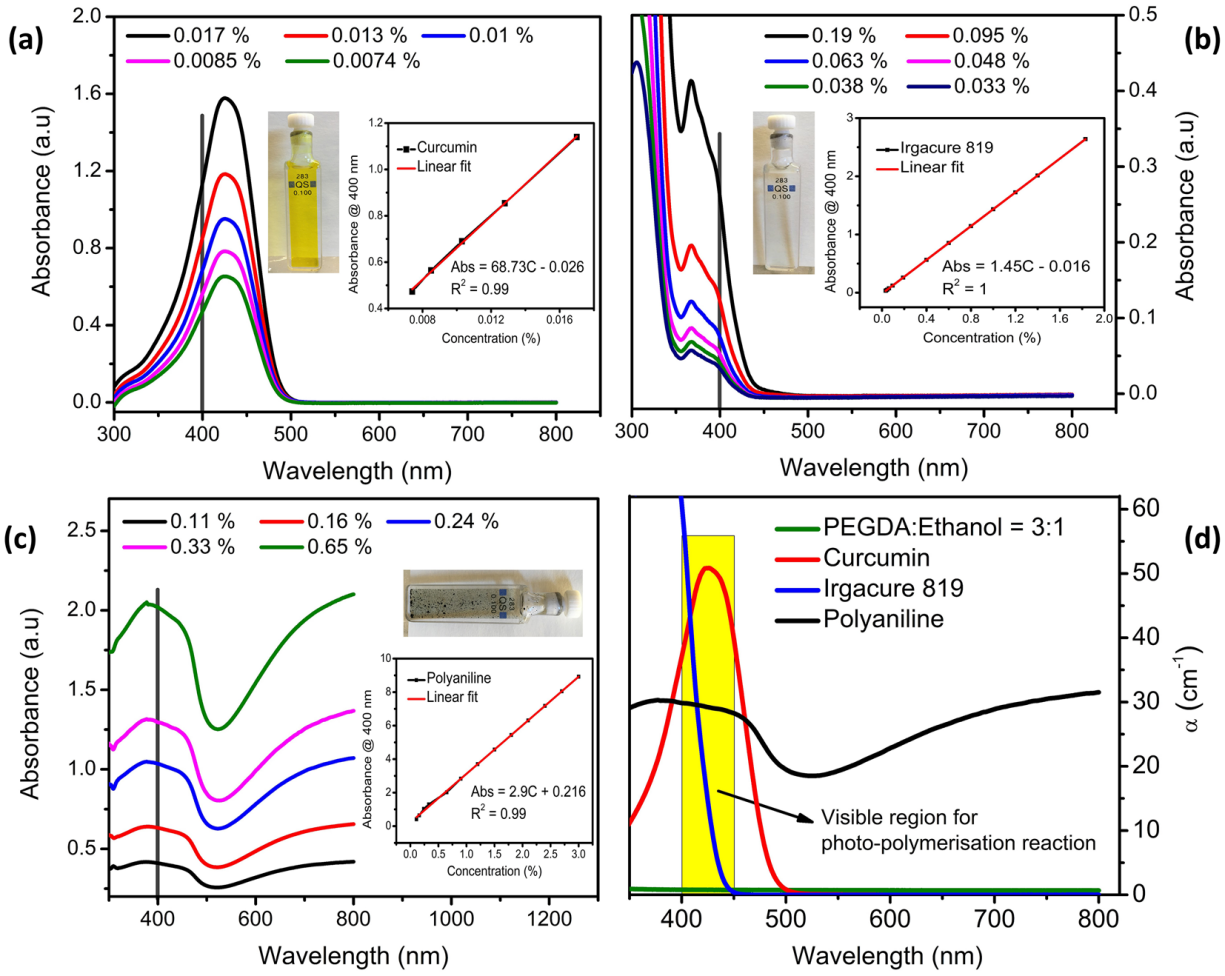
The absorbance spectra of (**a**) curcumin in ethanol, (**b**) Irgacure in acetone, and (**c**) PANI in PEGDA at low concentrations. The insets show the absorbance values at 400 nm plotted against these concentrations of each component to obtain a line graphs. These extrapolated line graphs were then used to estimate the values of absorbance used in the actual synthesis. (**d**) The absorption coefficient in 300–800 nm wavelength range of all components relative to their actual concentration in the final solution.

The efficiency of the photo-polymerization reaction strongly depends on the balance of the light-absorbing capacity of the individual components in the precursor solution. This capacity can be described in terms of absorption coefficient (α) which tells how much light gets absorbed per centimeter of the solution while travelling through it. To calculate it, first, the absorbance vs wavelength spectra of the components were obtained at lower concentrations using a spectrophotometer as shown in Fig. 2a–c. The direct measurement of these spectra relative to the actual concentration in the solution could not be obtained since UV–Visible spectroscopy does not work well with concentrated solutions.

After obtaining multiple absorbance vs wavelength spectra at low concentrations, the absorbance values at the 400 nm cut-off were plotted against the corresponding concentrations to obtain the absorbance vs concentration line graphs for all components. This line graph can also be obtained at any wavelength since the absorption proportion will remain the same.

The line graphs were extrapolated to estimate the absorbance values relative to the actual concentrations in the solution and these values were substituted in Eq. (4) to calculate the absorption coefficient for all components at 400 nm wavelength:

4 $$\alpha =2.303 \frac{A}{d}$$

where A = absorbance values from the line graph, d = path length = 0.1 cm (thickness of the cuvette).

The absorption coefficients were calculated within the 300–800 nm wavelength range and plotted in Fig. 2d, showing the relative absorption power of curcumin, Irgacure and PANI in the precursor solution. This sense of competition among the individual components to absorb light allowed us to find their optimal concentration in the final solution. For example, (i) the concentration of Irgacure should be high enough to generate free radicals to crosslink the solution, (ii) curcumin should have enough concentration to avoid light from penetrating deep into the solution but at the same time should be the least possible to allow the generation of free radicals without absorbing much of light energy, (iii) PANI should also not absorb most of the incoming light but it should be enough to impart electrical conductivity to the highly inert PEGDA matrix. In our custom-built projection micro-stereolithography setup, the photo-polymerization reaction took place within the 400–450 nm range referred to as the “visible region for photo-polymerization reaction”. Beyond this range, Irgacure does not absorb any light to produce free radicals for crosslinking as shown in Fig. 2 (bottom right). Generally, curcumin absorbs light strongly compared to Irgacure at the same concentration. Therefore, in the final solution, its concentration was kept much lower (0.016%) compared to the concentration of Irgacure (1.83%).

### 3D printed scaffolds

**Figure 3:**
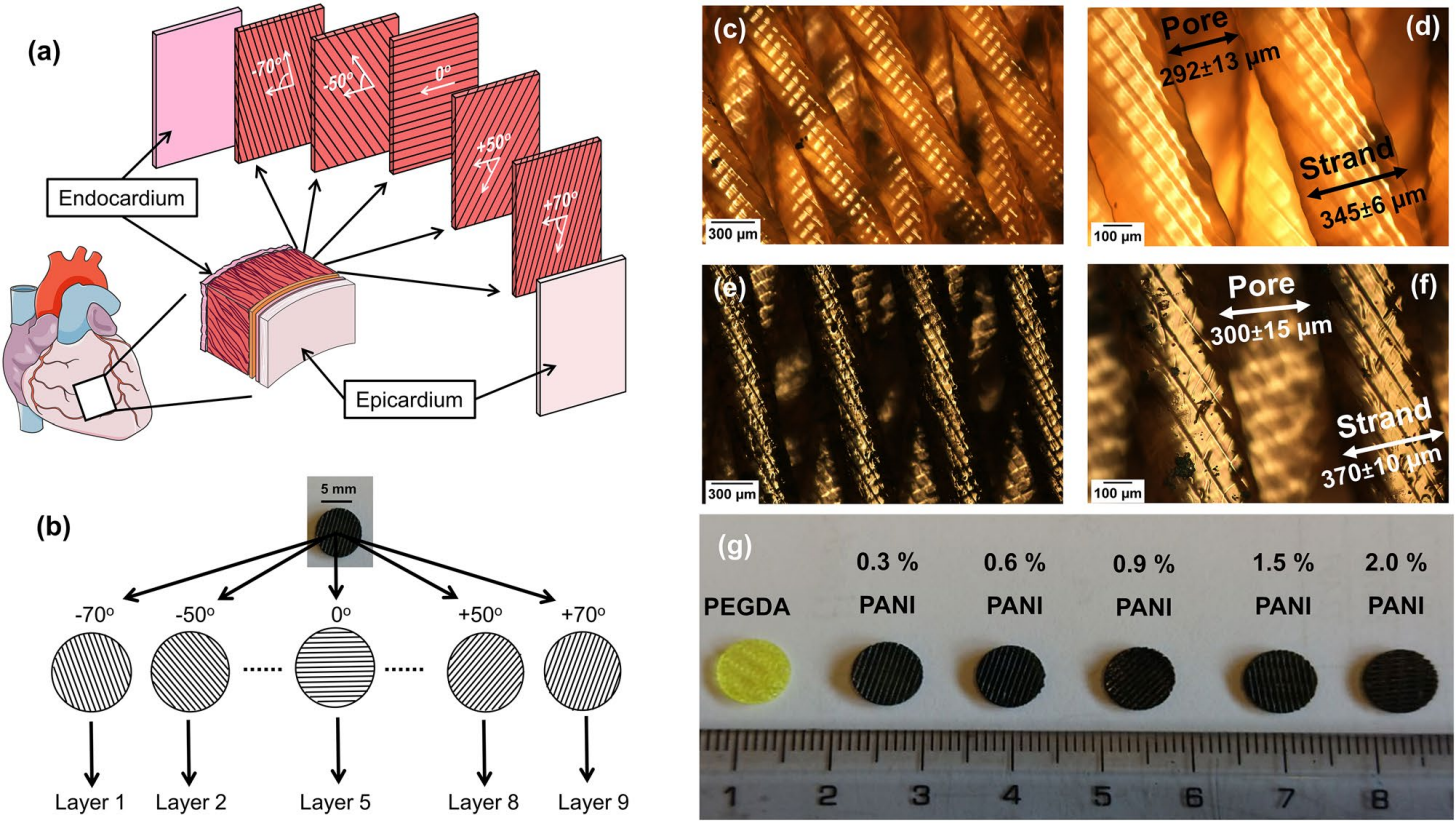
Structure and morphology of the printed scaffolds. (**a**) In the myocardium the myofibers are oriented at different angles in a hierarchical manner. (**b**) Different layers aligned along these angles were stacked on top of each other to obtain a 3D scaffold mimicking this structure. (**c**) Non-conductive PEGDA scaffold at 5X and (**d**) at 10X, (**e**) conductive scaffold (0.3% PANI) at 5X and (**f**) at 10X. (**g**) Scaffolds with different concentrations of PANI. Figure 3A was made using Servier Medical Art under the CC-BY 3.0 license.

The orientation of the myofibers changes along different angles through the thickness of the myocardium (Fig. 3a). Myofibers rotate from + 70° to + 50° in the sub-epicardial region, to 0° in the mid-wall and to − 50° to − 70^o^ in the sub-endocardial region^26^. To mimic this versatile bio-architecture, the strands in each printed layer were aligned along these angles as shown in Fig. 3b. Deposition of these 2D layers on top of each other created a 3D scaffold. Figure 3c–f shows the optical images of the 3D-printed scaffolds. In this study the distance between two strands is stated as “pore size” according to the pore size classification consistent with the international prefixes system^27^. Non-conductive PEGDA scaffolds had an average strand diameter of 345 ± 6 µm, with a pore size of around 292 ± 13 µm as measured using ImageJ software. While the conductive scaffolds had an average strand diameter of 370 ± 10 µm with an average pore size of 300 ± 15 µm. The homogenous distribution of the PANI phase inside the scaffolds gave them the characteristic dark color (Fig. 3g) and imparted semi-conductive properties to the highly inert PEGDA matrix. The electric current can flow through the PANI chains via the electron hopping mechanism i.e., when electrically stimulated, the free electrons can jump from one polaron/bipolaron (native to the doped PANI) to another. The details of the conductivity mechanism are given elsewhere^28^.

**Figure 4:**
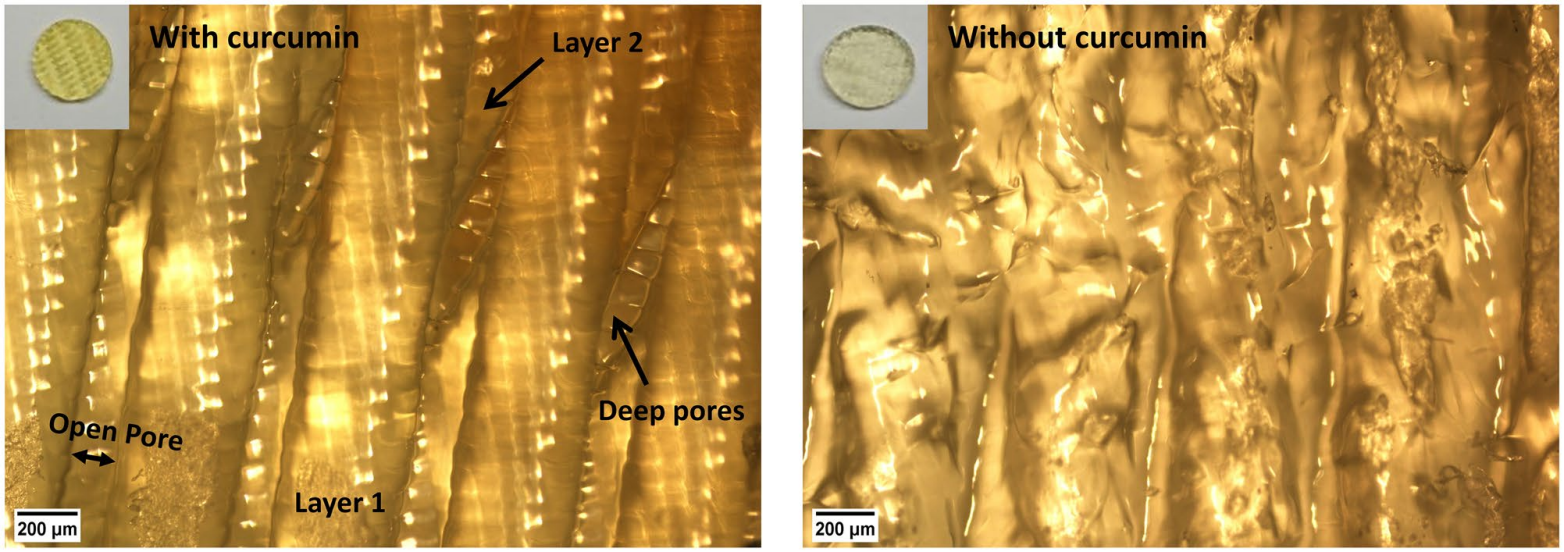
Role of curcumin in our custom-built 3D printing setup. Scaffold demonstrated well defined morphology and open pores with curcumin in the precursor solution (left, 5X). In the absence of curcumin, the layers diffused into each other leading to poor scaffold morphology and closed pores (right, 5X). Characteristic yellow color can be distinguished between scaffolds due to curcumin in it (top left).

Curcumin was used as a liquid filter in this 3D printing process. It prevented light from penetrating deep into the solution to reach the lower printed layers and made sure only the top of the solution received enough light energy for the photo-polymerization reaction to take place. In the absence of curcumin, the light penetrated deep into the solution producing scaffolds with closed pores and poor morphology as shown in Fig. 4.

### Scanning electron microscopy

**Figure 5:**
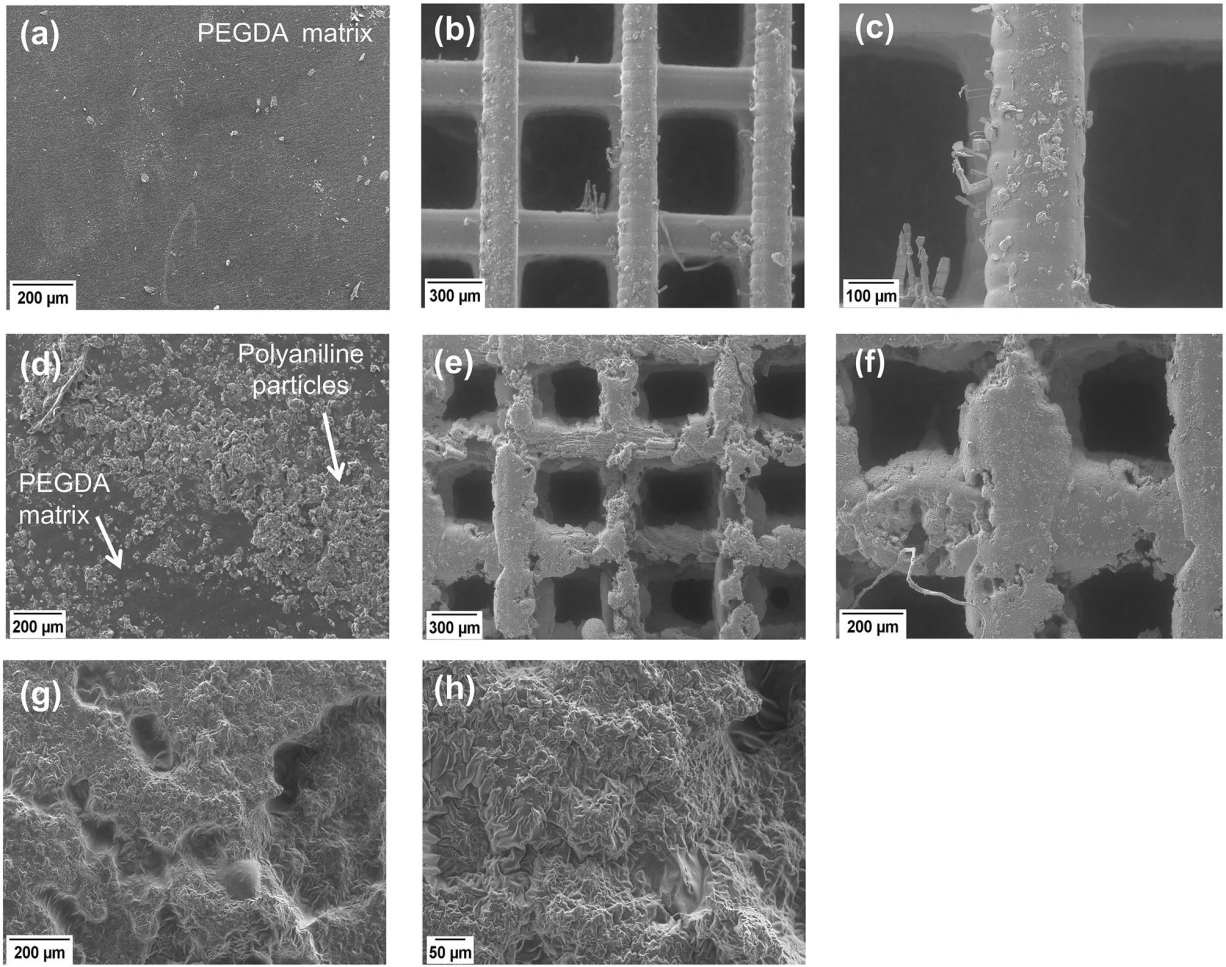
Morphology of the printed scaffolds. (**a**) Matrix of PEGDA without PANI, (**b**) Non-conductive PEGDA scaffold at 60X and (**c**) at 150X, (**d**) 2% PANI embedded in the PEGDA matrix, (**e**) conductive scaffold with 2% PANI at 50X and (**f**) at 100X, (**g**) wrinkly morphology indicates the homogeneous distribution of 3% PANI in the PEGDA matrix at 100X, and (**h**) at 200X magnification.

Scanning electron microscopy was performed to evaluate the morphology of the printed scaffolds. As it can be seen in Fig. 5a–c in the absence of PANI in the precursor solution, the obtained non-conductive scaffolds had well-defined geometry and pore size due to the optical transparency of the solution. Conversely, the PANI containing solution made the printing more complex since PANI also starts to absorb the incoming light as shown in Fig. 2 (bottom right). This made the photo-polymerization reaction inefficient to some extent depending on PANI concentration and the resulting scaffolds had somewhat rough geometry with an irregular pore size as shown in Fig. 5d–f. To tackle this, the solution was exposed for longer durations per impression as shown in Table 1. Beyond 2.0% PANI concentration, the printing of a scaffold with well-defined geometrical features was quite challenging. It can be associated with the fact that the absorption coefficient (α) of PANI at these concentrations was high enough to prevail over the light-absorbing ability of other components that made the solution crosslinking difficult. Due to this, no free radicals were produced at this concentration to crosslink the solution. Furthermore, at 2.5% and 3.0% PANI concentrations, the solution became too viscous to work with. Even after exposure for more than 20 s per impression a well-defined scaffold with proper morphology and pore size was never achieved. Only a solid disc was obtained at these concentrations which was further characterized using electron microscopy as shown in Fig. 5g–h and other techniques.

**Figure 6:**
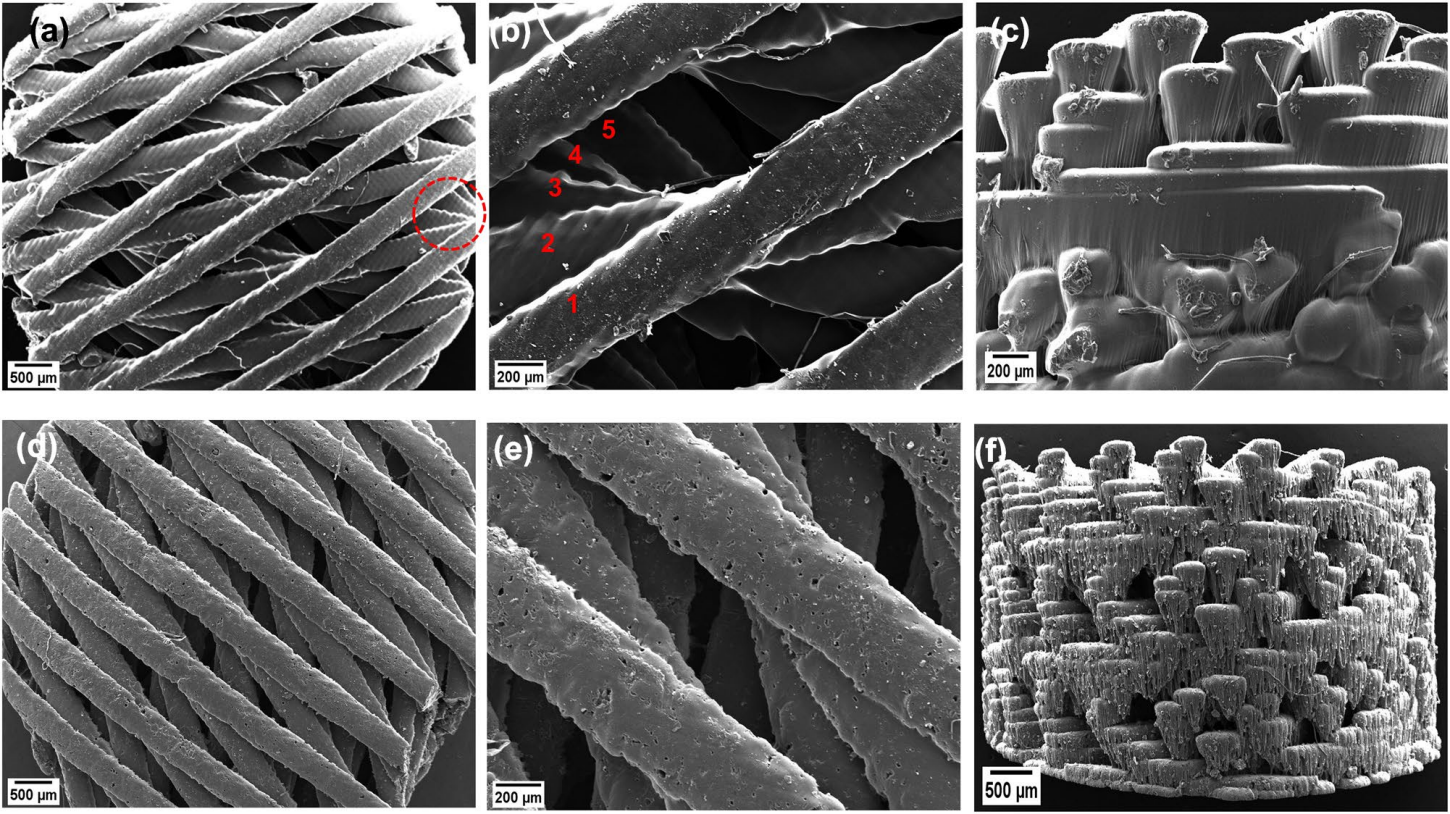
Hierarchical morphology of the printed scaffolds. (**a**) PEGDA scaffold at 44X while the circle indicates strands rotation deep into the scaffold, and (**b**) PEGDA scaffold at 133X while numbers indicate layer 1 on top and the subsequent layers deep into the scaffold rotating at certain angles, (**c**) cross-section of the at 107X, (**d**) PEGDA scaffold with 2.0% PANI at 44X, and (**e**) at 133X, (**f**) cross-section at 67X representing various layers deposited on top of each other during 3D printing*.*

The hierarchical morphology of the scaffold was also evaluated using a deeper SEM analysis. Figure 6a–c corresponds to PEGDA scaffolds without PANI, while in Fig. 6d–f the PEGDA-PANI scaffold has 2.0% PANI. It can be seen that the strands rotate at different angles indicated by the circle in Fig. 6a. A more magnified image (Fig. 6b) shows the rotation of various strands deep into the scaffold indicated by numbers that correspond to the layer numbers in Fig. 3b. The cross-sectional view of the scaffolds can be seen in Fig. 6c,f in which the strands travel into the plane of the paper. It can be seen in these Figures that the strands were deposited on top of each other while aligning at different angles confirming the hierarchical morphology of the scaffolds. The maximum concentration of PANI at which printing was possible using our custom-built PμSL printer was 2.0%. That is why a lot of PANI particles can be seen in Fig. 6f just like these particles can be seen embedded in the PEGDA matrix in Fig. 5d. Contrary to this, scaffold without PANI had well-defined morphology as shown in Fig. 6c, 5b and 5c.

### Infrared spectroscopy

**Figure 7:**
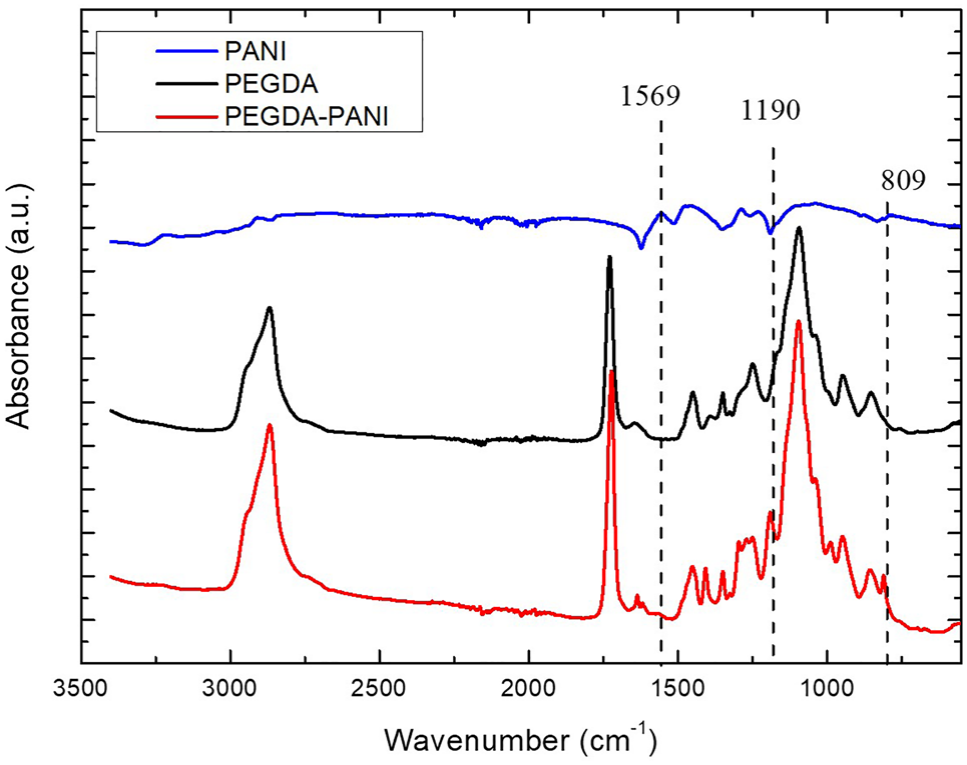
FTIR-ATR spectrum of conductive (emeraldine salt) form of PANI (blue), PANI embedded in PEGDA (red) and PEGDA (black).

PANI exists in three redox states: fully reduced leucoemeraldine, partially oxidised emeraldine, and fully oxidised pernigraniline^29^. These structures may be protonated or deprotonated, whether in an acidic or basic environment, respectively. Without protonation, these forms are referred to as “base” such as emeraldine base. After protonation, they are referred to as “salt” such as emeraldine salt. Emeraldine salt is the only electro-conductive form of PANI. Infrared spectroscopy provided information about the oxidation states of PANI chains. The analysis of the vibrational spectrum pointed out the protonated emeraldine form of PANI achieved during the synthesis process (Fig. 7).

In particular, in the spectra of PANI and PEGDA-PANI composite (blue and red line, respectively), we can observe the peaks at 809 and 1569 cm^−1^, representing the C–H bending vibration mode of the benzene ring and the C=C stretching of the quinoid ring^30,31^. While the peak at 1226 cm^−1^ in the PANI spectrum, shifted to 1190 cm^-1^ in the PEGDA-PANI can be attributed to the C–N stretching mode in the benzenoid group^32^. These distinctive signals confirm the successful synthesis of the PANI in the conductive form, i.e., the emeraldine salt. The characteristic PEGDA peaks can be found at 1095, 1723 and 2868 cm^-1^ in both the PEGDA and PEGDA-PANI spectra (black and red line, respectively). These signals can be respectively ascribed to the stretching vibration of the –C–O–C–, –CH and –C=O of the PEGDA backbone^33^.

### Electrical conductivity

The two-probe technique was used to measure the bulk electrical conductivity of printed scaffolds. Table 2 reports the values obtained for the PEGDA-PANI scaffolds compared to those of murine myocardial tissue and the inert PEGDA scaffold. It can be noted that PEGDA is characterized by a conductivity in the range of 10^–9^ S/m typical of plastic insulators. However, the introduction of PANI in this inert scaffold increased the conductivity by 10^3^ fold up to ~ 10^–6 ^S/m thus imparting a semi-conducting behavior. This value is comparable to those obtained for PEGDA-PANI materials with the same filler amount^34^. The improvement in conductive properties follows the increase of PANI concentration indicating that a more efficient charge transfer among PANI chains was accomplished by the formation of a homogeneous conductive network inside the PEGDA matrix. No electrical response was detected below 0.9% PANI content. This could be due to a lack of bulk conductive networks among the polymer chains.

**Table 2:** Electrical conductivity of 3D printed scaffolds measured using the two-probe technique.

Sample	Electrical conductivity (S/m)	References
Murine myocardium	0.16	^35^
PEGDA	(7.6 ± 0.3) × 10^–9^	^34^
0.3% PANI	–	Present work
0.6% PANI	–	Present work
0.9% PANI	(2 ± 2) × 10^–6^	Present work
1.5% PANI	(2.9 ± 0.9) × 10^–6^	Present work
2.0% PANI	(3.8 ± 0.1) × 10^–6^	Present work
2.5% PANI	(5.0 ± 0.6) × 10^–6^	Present work
3.0% PANI	(9.8 ± 0.4) × 10^–5^	Present work

### Cyclic voltammetry

**Figure 8:**
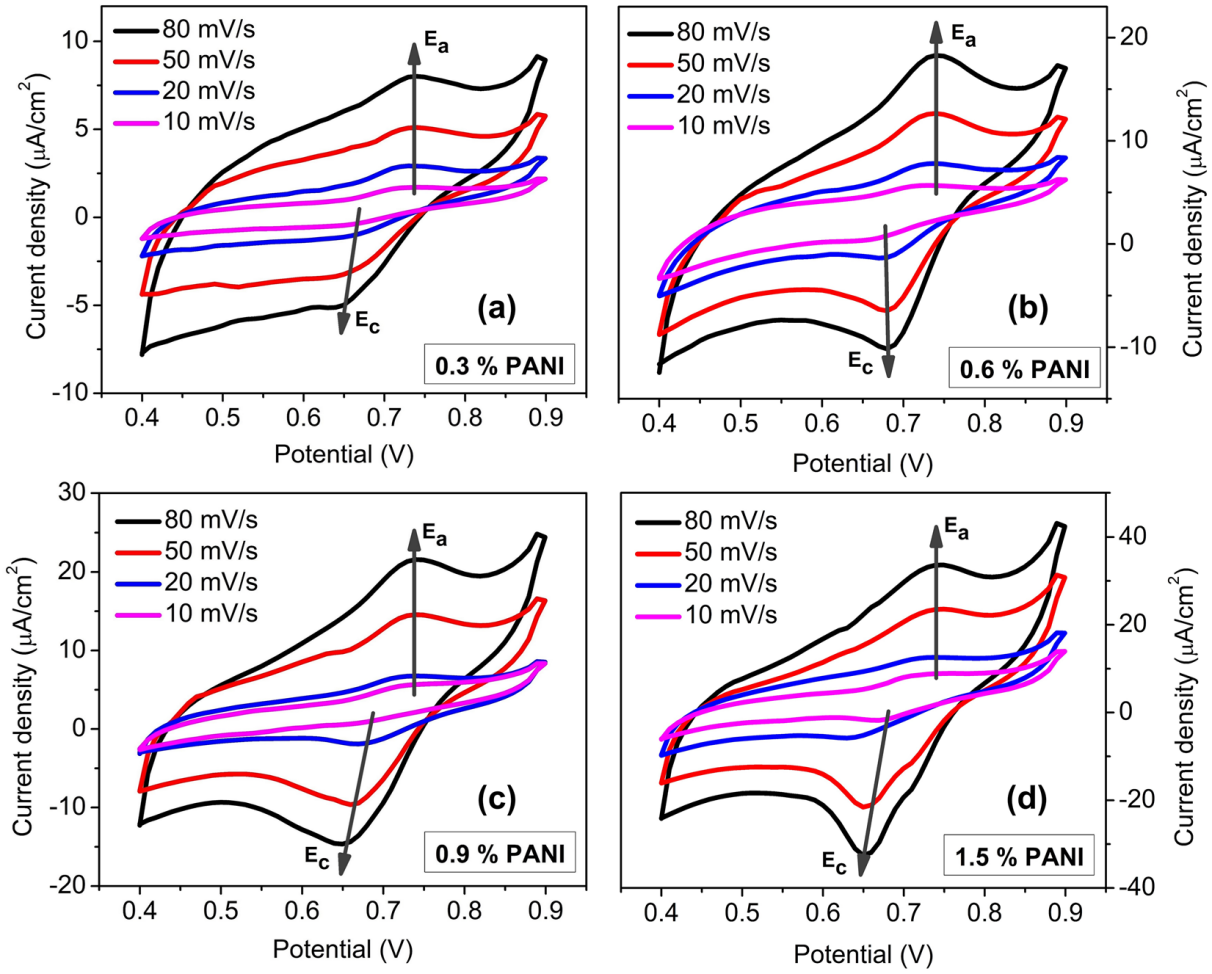
Cyclic voltammograms of conductive scaffolds at different scan rates of (**a**) 0.3% PANI, (**b**) 0.6% PANI, (**c**) 0.9% PANI and (**d**) 1.5% PANI.

Cyclic voltammetry (CV) measurements were performed to test the electrochemical activity of the produced scaffolds. The analysis was carried out in 0.1 M HCl solution, and the data were acquired at different scan rates. It is evident from Fig. 8 that the CV curves show broad anodic (E_a_) and cathodic (E_c_) peaks at around + 0.74 V and + 0.66 V, respectively. This redox couple can be related to the transition from the PANI emeraldine semioxidized form to the pernigraniline completely oxidized form and vice versa^36,37^. The presence of such an electrochemical feature indicates the chemical reversibility of the redox processes. Moreover, an increase in the current density was observed with the increase of PANI concentrations in the PEGDA matrix according with what found for the electrical behavior. It is interesting to note that the scaffolds with 0.3% and 0.6% PANI are able to respond to the electrical stimulation while performing the CV, while it is not possible to measure their conductivity values via the two-probe method. These results could be clarified considering that the electrochemical processes not only involve electron transfer but also ionic diffusion between the polymer and the electrolyte solution.

As it can be observed in Fig. 8, an increase in the current density with a scan rate ranging from 10 to 80 mV/s was found. While the anodic peaks (E_A_) retain their position, the cathodic (E_C_) ones are slightly shifted to lower potential. This feature can be associated with the partial electrochemical irreversibility of the chain reduction probably due to a complex kinetics of the process. It could also be due to some chemical/structural modifications accompanying the electron transfer^22^. In brief, the analysis of the electrochemical investigations highlighted the electroactivity and the ability of PANI inclusion to modulate the ionic concentration into the hydrogel PEGDA matrix. In this way, this feature can be exploited efficiently in several biomedical fields to produce devices/scaffolds mimicking the electrical nature of biological systems.

### Swelling degree

**Figure 9:**
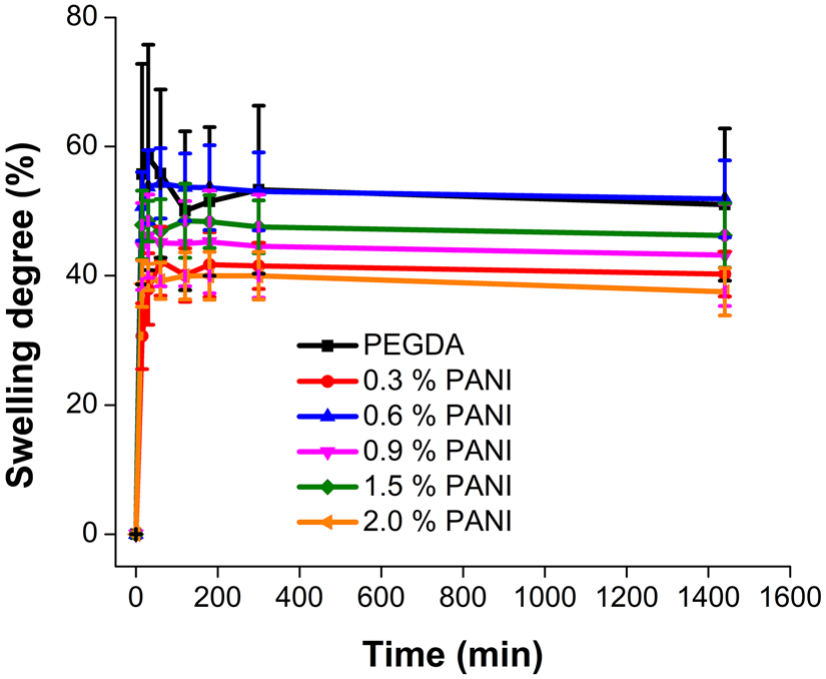
Swelling degree of 3D printed scaffolds with different concentrations of PANI.

The swelling degree was measured by immersing the scaffolds in the 1X PBS at 37 °C for 24 h. As shown in Fig. 9, the maximum swelling degree of the scaffolds (≤ 60%) is unpronounced compared to the typical hydrogels that usually demonstrate abrupt swelling behaviors. The cultured cells exposed to abruptly expanding environments can often trigger apoptosis due to high stresses generated in the expanding hydrogel network^38^. In our case, all scaffolds demonstrated somewhat steady swelling behavior and reached their limit within 30 min of immersion in the PBS. The presence of PANI inside the PEGDA matrix decreased the swelling extent regardless of its concentration. This can be due to a probable higher rigidity of the composite material with respect to a pure PEGDA hydrogel scaffold.

### Hydrolytic degradation

The degradation of the printed scaffolds was investigated by immersing the scaffolds in 1X PBS at 37 °C for 4 weeks since it is well known that the ester bonds (–O–C=O), usually formed during the PEGDA hydrogels radical crosslinking, are prone to hydrolysis reactions^39^.

**Figure 10:**
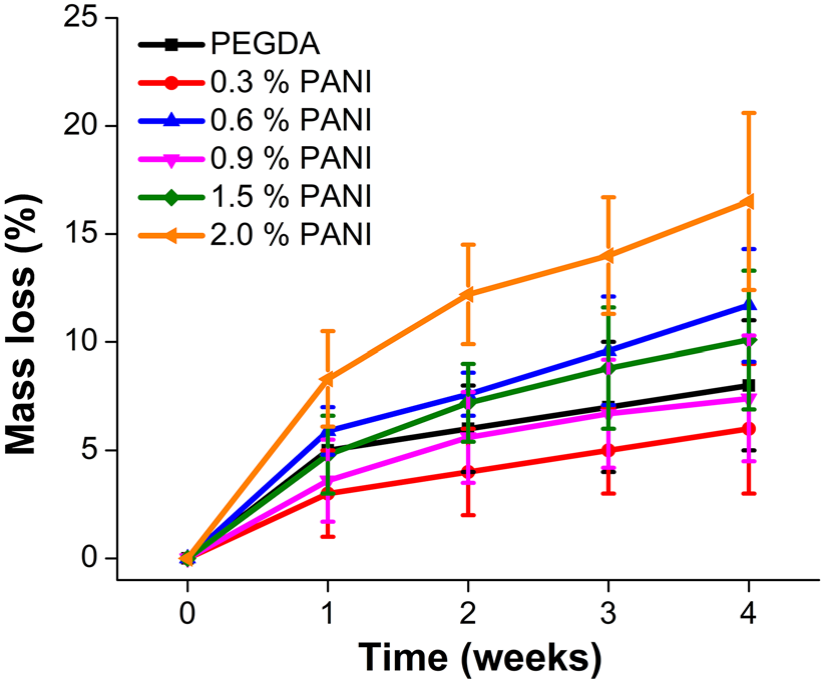
Hydrolytic degradation profile of 3D printed scaffolds with different concentrations of PANI.

As we can see in Fig. 10, for low concentrations there is no significant correlation between the degradation process and the presence of PANI within the PEGDA matrix within the experimental error. A more consistent degradation is recorded at 2% PANI inclusion, reaching a 15% mass loss after four weeks. This can be associated with a lower PEGDA crosslinking reasonably induced by the higher presence of PANI in that sample.

### Cell viability

**Figure 11:**
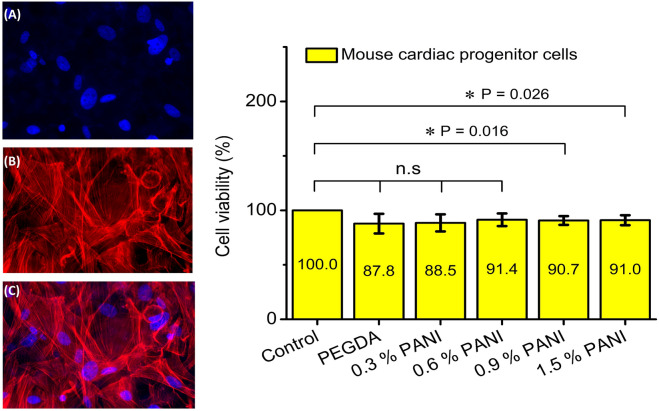
Viability of the mouse cardiac progenitor cells (mCPCs) on the printed scaffolds (n = 6). On the left, the representative micrographs show mCPCs stained with (**A**) DAPI (nuclei in blue), (**B**) F actin (red) and (**C**) their merge. On the top right, the cell viability was assessed via Trypan Blue assay after 5 days of culture. Bars represent the viability of the cells relative to the percentage of the control. Data are represented in Mean ± S.D of the triplicate experiments. Two tailed t-test with equal variance was performed to find out the statistical significance (*P < 0.05) between the control group (cell suspension) and the other groups (cell suspension + scaffold) while one way ANOVA between groups resulted in statistically insignificant results (*P* = 0.23) showing that the mean viability was the same among all groups as can be observed by the values indicated in the bar graph.

The viability of mouse cardiac progenitor cells (mCPCs) was evaluated by Trypan Blue assay after 5 days of incubation as shown in Fig. 11. The control group for this experiment was the cell suspension placed in different wells of a 24-well plate without any scaffold in the wells. Results demonstrated that the scaffolds did not induce profound cytotoxic effects impeding cellular growth relative to the control group. Indeed, all scaffolds demonstrated good biocompatibility and the viability was not much different compared to the control group. However, the electrical nature of the conductive scaffolds seemed to be improving the viability compared to non-conductive scaffolds although the results were not statistically significant as the mean viability of all groups appears to be the same (*P* = 0.23 between groups, one-way ANOVA). The conductive scaffolds may have led to better intercellular communication thus improving the relative viability due to their electrical nature, a characteristic of the conductive scaffolds observed in previous studies^40–42^. This could also be a reason for the existence of statistical significance (via t-test) between the control and high concentration PANI scaffolds while low PANI concentration or pure PEGDA scaffolds did not result in statistical significance due to the absence of intercellular communication. These observations show that PEGDA-PANI scaffolds could be a better alternative to traditional non-conductive scaffolds when it comes to regenerating highly conductive tissues.

## Discussion

The holy grail of tissue engineering has always been to exactly mimic the characteristic features of the intended tissue to engineer. These features include, but are not limited to, tissue bio-architecture, elasticity/stiffness, biodegradability, and biocompatibility. A major role in this endeavor is played by the scaffold, a polymeric structure designed to support cellular growth and differentiation, while emulating the ECM structure and function. Significant progress in scaffold design has been made when relatively simple organs, such as blood vessels, skin, bladder, and cartilage, were engineered^43–47^. Myocardium, instead, is very difficult to engineer due to its 3D anisotropy, baffling biomechanical properties, and complicated micro/nano bio-architecture.

So far, cardiac scaffolds have been manufactured using biologically inert materials to be a mere support for cell growth. Instead, the myocardial ECM, which is the natural scaffold of the myocardium, is a scaffold material produced by the fibroblasts and made of a jelly-like substance (matrix) and fibers of different lengths and stiffness, and can address cell fate. Quiescent fibroblasts are dynamically distributed within the myocardium, while pathological disorders trigger their activation to myofibroblasts subverting the ECM composition and accumulation and hence the myocardial function^48^. The ECM delivers biological (growth factors, cytokines, hormones, etc.) and physical signals (as mechano-structural factors, stiffness, nano rugosity, micro-porosity, etc.) concurring to modulate the cardiac cell fate. Besides biological signals, particular attention has been paid so far to deciphering how cells translate mechanical stimuli into intracellular signals, while only a few studies have focused on the cell mechanism sensing electrical signals (electro sensing).

Several studies evidenced that bio-instructive materials, endowed with both bioelectric and topographical cues, represent a promising approach to regenerate highly anisotropic tissues such as skeletal muscle. In^49^, the nanofiber alignment and PANI concentration in electrospun polycaprolactone scaffolds were found to induce myotube formation in myoblasts, confirming the synergistic effects of electrical and topographical cues. However, the in-depth mechanisms through which electroconductive materials instruct cells remain to be understood. Other studies^50–54^ have demonstrated the effectiveness of electroconductive scaffolds, but no conclusive evidence has been so far reported on the role of their electrical conductivity. Indeed, electrical signals weaker than the action potential were extracellularly detected using the multielectrode array technology^55–59^ but, very likely, their action alone is not sufficient to determine the cell fate. The beneficial effects of electroconductive constructs were also observed when implanted at the injury site in rat MI models^60–63^. Nevertheless, these studies were mostly aimed at repeating the characteristics of the mature myocardium to fill the conductivity gap created by the post-infarction scars, where the rational was just to re-create a guidance for the electric impulse presiding over myocardial contraction. An innovative idea could be to develop a system for identifying and characterizing microcurrents potentially not involved in contraction, but relevant (together with other physical and biological signals) in cell–cell communication to determine the cell fate. In this context, we proposed a preliminary technical study for developing electroconductive scaffolds for investigating the electro-sensing of immature cardiac cells. The optimization of high resolution micro-stereolithography process has led us to obtain electroconductive 3D printed PEGDA-PANI systems. Different concentrations of PANI allow to modulate the electroactivity and electroconductivity in a way that the electrical and mechano-spatial signals can be reciprocally tuned to deliver stimuli to immature cells. These achievements are prominent in view to determine cell fate and sustain their final phenotype. In this respect, the performed cell viability tests demonstrate that PEGDA-PANI materials do not induce severe cytotoxic effects and confirm their validity as scaffolds for a more in-depth understanding of the biological and physical signals governing cells development.

## Conclusion

Projection micro-stereolithography proved to be a useful tool for mimicking the striated bioarchitecture of the heart tissue. Scaffolds, even though semi-conductive, have shown their biological efficacy when cultured with mouse cardiac progenitor cells. It shows that within the reference frame of immature cardiac stem cells, the semi-conducting scaffolds may improve the intercellular communication and synchronization of developing/immature cells.

Indeed, the development of scaffolds as electrically conductive as myocardium (0.16 S/m) is highly desirable to manufacture engineered cardiac tissues, and, eventually, to restore the lost electrical functions of the injured heart. However, it is still a challenge to develop such constructs utilizing current technology. Hence, it can be concluded that electrically conductive scaffolds could provide better platforms when it comes to engineering highly conductive biological tissues compared to non-conductive scaffolds. However, new alternatives to conducting polymers that would be biodegradable and can demonstrate high electrical properties must be found to push the current conductive scaffolds from being semiconductors to good/high conductors.

## Data Availability

The authors declare that the relevant datasets supporting this study are given within this article. However, the raw datasets supporting various findings can be requested to the corresponding author.
